# Cellulose Degradation by Calcium Thiocyanate

**DOI:** 10.3390/polym11091494

**Published:** 2019-09-12

**Authors:** Myung-Joon Jeong, Sinah Lee, Bong Suk Yang, Antje Potthast, Kyu-Young Kang

**Affiliations:** 1 Department of Wood Science and Technology, Chonbuk National University, Jeonju 54896, Korea; mjeong@jbnu.ac.kr; 2 Department of Biological and Environmental Science, Dongguk University–Seoul, Goyang 10326, Korea; sssssina1@gmail.com (S.L.); ybs95@dongguk.edu (B.S.Y.); 3 Department of Chemistry, BOKU-University of Natural Resources and Life Sciences, Vienna, Konrad-Lorenz-Str. 24, A-3430 Tulln, Austria; antje.potthast@boku.ac.at

**Keywords:** cellulose aerogel, calcium thiocyanate, cellulose hydrolysis, cellulose oxidation, Brunauer–Emmett–Teller (BET), size exclusion chromatography–multi-angle light scattering system (SEC–MALS)

## Abstract

The dissolution process of cellulose aerogels is an important part of their production. However, if the cellulose is severely degraded during the dissolution process, the quality may be low. To evaluate the degradation of cellulose during the dissolution process using calcium thiocyanate, the hydrolysis and oxidation of cellulose were evaluated by the change in absolute molecular weight and by the changes in the content of carboxyl and carbonyl groups introduced into the cellulose hydroxyl group, respectively. A noteworthy hydrolysis phenomenon was found in the cellulose dissolution process. The rate of hydrolysis increased as the number of hydrates in calcium thiocyanate decreased and as the reaction temperature increased. In the case of the reaction with calcium thiocyanate containing six hydrates, the time to reach a 50% loss of the degree of polymerization of cellulose reduced from 196 to 47 min as the reaction temperature was increased from 100 to 120 °C; however, the effect on oxidation was not significant. The Brunauer–Emmett–Teller (BET) surface area reduced as the degree of cellulose polymerization decreased. Therefore, it is necessary to consider how the cellulose degradation occurring during the cellulosic dissolution process can affect the quality of the final cellulose aerogels.

## 1. Introduction

Fossil fuel-based materials replaced many traditional materials during the last century [1]. Owing to global warming, recent environmental demands resulted in renewed interest in the use of renewable natural resources as direct sources of biopolymers [2]. Among the various natural polymers, this study focused on cellulose, which is widely used as pulp for making paper, film, and bio-plastic materials. Cellulose is an abundant natural polymer that is polymerized with β-1,4-glycosidic bonds of d-glucose. The degree of polymerization (DP), from under 1000 for chemically pulped fibers to over 10,000 for natural fibers, depends on the type of cellulose resource and the pulping process [3]. Because of these advantages, various studies on topics, such as environmentally friendly cellulose [2] and nanocellulose [4] in the form of various derivatives for nanofiber [5], biomedical [6,7,8], functional film [9], sensor [10,11] and purification [12,13,14] applications, were conducted to utilize cellulose from a wide range of different sources [15].

The production of cellulose aerogel is a valuable research area for the effective utilization of natural cellulose [16,17,18,19]. Cellulose aerogels are ultra-lightweight, porous solid materials with high specific surface area, and their ranges of density, porosity, and specific surface area are 0.0005–0.35 g/cm^3^, 84.0–99.9%, and 10–975 m^2^/g, respectively [20]. Currently, they are applied to thermal insulation materials [21,22,23], adsorption materials for organic solvents and water purification [24,25,26,27], biomedical applications such as drug release control [28], bacterial adsorption [29], and scaffolds for three-dimensional (3D) cell cultures [30]. In other words, cellulose aerogels are generally multifunctional and environmentally friendly materials. 

These cellulose aerogels are generally prepared through the processes of dissolving the cellulose, preparing the sol–gel (wet gel), and drying the cellulose wet gel [20]. The process of dissolving the cellulose is essential for the preparation of cellulose aerogels via a cellulose wet gel. However, each cellulose chain forms micro- and macrofibrils owing to the intramolecular and intermolecular hydrogen bonding between neighboring anhydroglucose units in the glycosidic bond area. The hydrogen bonds in the formed cellulosic bundle hinder the dissolution of the cellulose; thus, this is a limiting factor in various applications, including that of cellulose aerogels. To overcome these difficulties, various solvents systems were developed, which can destroy the micro- and macrofibril structure, and these solvents increase the amount of cellulose available, enhancing its potential for new kinds of applications. 

Two main types of cellulose dissolution methods were studied: the first is an indirect dissolution method in which cellulose is dissolved after derivatization, and the second is a direct dissolving method using solvents, which separates the individual cellulose chains from each other without derivatization. The indirect dissolving methods, such as nitration, xanthation, esterification, and etherification, are able to control the chemical structure and physical properties by derivatizing the hydroxyl groups in the cellulose. However, the development of new alternative dissolving methods is required because of difficulties in recovering the solvent, problems with the toxicity in products, and various environmental issues. In contrast, the direct dissolution methods do not require the derivatization of cellulose and most of the solvents can be recovered, which is advantageous in not only reducing the environmental implications but also improving the economic aspects. 

Typical solvents that dissolve cellulose directly include inorganic molten salt, ionic liquids, *N*-methylmorpholine *N*-oxide (NMMO), alkali–urea, and lithium chloride/*N*,*N*-dimethyl acetamide (LiCl/DMAc). Ionic liquids are salts with high solvation ability, which generally have very low vapor pressure with melting points below 100 °C, and the ions interact directly with the hydroxyl groups in the cellulose chain. However, industrial applications are still limited due to technical and economic issues [31,32]. NMMO is the only industrially commercialized solvent for cellulose that is used in the Lyocell process. The oxygen of the highly polar N–O group in NMMO molecules is able to form hydrogen bonds with cellulose, which is capable of breaking the hydrogen-bonded cellulose network [20,33]. In the case of alkali–urea solution, the alkali interacts with the hydroxyl group in cellulose molecules at low temperature; although urea has no strong direct interaction with cellulose, the urea prevents the re-aggregation of cellulose molecules due to the van der Waals force. As a result, the solubility and stability of the cellulose is improved. However, it can only apply to cellulose with a low DP, which limits the mechanical properties of products [34]. Lithium chloride/*N*,*N*-dimethyl acetamide forms a complexation between the LiCl/DMAc complex and cellulose molecules, which was only used as a laboratory cellulose solvent for measuring the molecular weight distribution and quantifying the carbonyl and carboxyl content [35,36] due to the high toxicity of DMAc. 

Inorganic molten salts were also used as solvents for cellulose with a wide range of DPs [37]. Various inorganic molten salts with hydrates were applied to dissolve cellulose without pretreatment. The status of inorganic salt hydrates is in between that of concentrated aqueous and solid states. The water molecules form coordinate covalent bonds with positively charged metal ions. Representative examples of the maximum coordination bound inorganic salt aqueous solutions are MgCl_2_·6H_2_O, LiCl·5H_2_O, LiClO_4_·3H_2_O, and ZnCl_2_·4H_2_O. Cellulose is dissolved in the following order: dispersion, dissolution, swelling, and dissolution in inorganic salts, which contain water molecules [38]. The advantages of inorganic salts include the fact that they can dissolve cellulose without any pretreatment of cellulose. The inorganic salt used in the dissolution process can be reused through a recovery process. The crystallization and morphological changes of the cellulose in the dissolution process, in which cellulose is dissociated by a coordination bond between the cation in the inorganic salt and the hydroxyl group in the cellulose, occur according to the composition of the inorganic salt. Cellulose dissolution using calcium thiocyanate, which is one of these inorganic salts, can break the hydrogen bonds between cellulose molecules through a coordination bond between the cationic (Ca^2+^) and hydroxyl groups in the cellulose molecules [39]. The cellulose aerogel prepared from a Ca(SCN)_2_·nH_2_O solution improved mechanical and dimensional stability due to its three-dimensional (3D) branched structure [40], and, as a result, cellulose aerogels prepared using this calcium thiocyanate method have excellent porosity. These dissolution characteristics of calcium thiocyanate are widely applied to the cellulose dissolving process for the manufacture of cellulose aerogels, which have potential applications as insulation materials, gas storage materials in fuel cells, filters for ultrafine particles, drug delivery systems, tissue scaffolds, and high-functional textiles [20,41].

The quality of cellulose aerogels can be affected by not only the cellulose solvent used for the dissolution process but also the kind of cellulose raw material source. From our previous study, another key aspect in the preparation of cellulose aerogels is that the DP of cellulose affects the aerogel’s pore structure and density [42,43]. Unfortunately, except for lithium chloride/*N*,*N*-dimethyl acetamide, all four types of solvents (i.e., ionic liquid, NMMO, alkali–urea, and inorganic molten salts) involve cellulose degradation during the dissolving process [40,44]. Unlike alkali–urea solution and NMMO, calcium thiocyanate can dissolve cellulose with a relatively high DP, which is more affected by its DP changes due to hydrolysis. In spite of various research studies on cellulose aerogel and its dissolving process, cellulose degradation during the dissolving process using calcium thiocyanate was not fully evaluated. Therefore, if cellulose degradation during the dissolving process could be minimized, the quality of cellulose aerogel’s pore structure and mechanical properties could be improved. In this study, we attempted to evaluate the influence of hydrolysis and oxidation in cellulose during the calcium thiocyanate dissolution process.

## 2. Materials and Methods

### 2.1. Materials

Whatman No. 1 paper sheets (GE Healthcare, Amersham, UK) comprising cotton linter (alpha cellulose content >98%) and α-cellulose powder (Sigma Aldrich, St. Louis, MO, USA) were used to prepare the cellulose aerogel. Calcium thiocyanate tetrahydrate (95%, Sigma Aldrich) and other chemicals used were of the highest grade commercially available. Solvents were of HPLC grade. Carbazole-9-carbonyloxy amine (CCOA) and 9*H*-fluoren-2-yl-diazomethane (FDAM) were not commercially available compounds and were synthesized in the laboratory according to procedures described in the literature [35,36].

### 2.2. Dissolution of Cellulose and Preparation of Cellulose Aerogel

Cellulose samples were mixed in calcium thiocyanate containing 6–10 hydrates. Then, the mixtures were dissolved at 100 and 120 °C for 30 and 60 min, respectively. The final concentration of the solution was controlled to 1.0% (*w*/*w*). The cellulose dope was poured into a cylindrical polypropylene tube for molding at approximately 80 °C. After cooling, the cellulose was regenerated by the solvent exchange process using ethanol. The cellulose wet gel was rinsed with fresh ethanol twice a day for three days.

Supercritical carbon dioxide drying was performed in a dryer equipped with a 300-mL autoclave (SF-1, Separex, Champigneulles, France). Drying was maintained at a constant supercritical carbon dioxide flow rate of 40 g/min for 3 h under 10 MPa and 40 °C. After the extraction was completed, the pressure in the autoclave was slowly reduced to 0.1 MPa/min to maintain an isotherm. 

### 2.3. Fluorescence Labeling of Functional Groups and Dissolution of Cellulose Aerogels

The CCOA labeling for carbonyl group profiling was performed according to methods described in previous studies [45,46]. Cellulose aerogel samples (25 mg) were agitated in a mixer for 20 s. The water was then removed by vacuum filtration. The samples were agitated in 4 mL of 20 mM zinc acetate buffer (pH 4.0) containing 5.0 mg of CCOA at 40 °C for a week.

The FDAM labeling for carbonyl group profiling was performed according to the method described by Bohrn et al. [47]. Cellulose aerogel samples (25 mg) were suspended in 0.1 M HCl and agitated in a mixer for 20 s. Then, the samples were washed with ethanol and DMAc successively and filtered. The DMAc was then removed by vacuum filtration. The samples were suspended in a mixture of 1 mL of FDAM solution with 3 mL of DMAc at 40 °C for a week. The final concentration of the solution was approximately 0.125 mol/L in DMAc.

After the reaction, the CCOA- and FDAM-labeled samples were washed and filtered. Then, the samples were activated with DMAc for a day after solvent exchange. The DMAc in the activated samples was then removed by vacuum filtration. The samples were dissolved in 2 mL of DMAc/LiCl (9%, *w*/*v*) at 23 °C for 1–2 days. The solution was diluted 1:3 with DMAc, filtered through a 0.45-µm filter, and injected into the size exclusion chromatography–multi-angle light scattering system (SEC–MALS (Wyatt Dawn DSP, Santa Barbara, CA, USA).

### 2.4. SEC–MALS Analysis

The hydrolysis and oxidation characterizations of cellulose aerogels were performed by measuring the molecular weight distribution and carbonyl and carboxyl content using the SEC–MALS system. This system was connected to fluorescence (TSP FL2000, CCOA: 290 nm excitation, 340 nm emission; FDAM: 252 nm excitation, 323 nm emission), MALS (Wyatt Dawn DSP), and refractive index (RI) detectors (Shodex RI-71, New York, NY, USA). The RI increment was 0.136 mL/g for cellulose in DMAc/LiCl (0.9%, *w*/*v*) at 25 °C and 488 nm. The separation was conducted on a set of three SEC columns (PL gel-mixed AALS, 20 µm, 7.5 × 300 mm). The mobile phase was DMAc with LiCl 0.9% (*w*/*v*). The molecular weight distribution and related polymer-relevant parameters were calculated using ASTRA and GRAMS.

During hydrolysis, cellulose can generate novel reducing end-groups (*REG*s) without the additional introduction of carbonyls by oxidation. The pure carbonyl content excluding *REG*s was calculated from the total carbonyl content (keto and aldehyde functionalities) and *REG* (Equation (1)). *REG*s were obtained from the number average molar mass (g/mol) according to Equation (2).

*Pure carbonyl content* (µmol/g) = *Total carbonyl content* (µmol/g) − *REG* (µmol/g); (1)

(2) $$REG\;(µ\mathrm{mol}/g)\;=\;\frac{1}{M_{n}}\times 10^{6}.$$

The hydrolysis kinetics of cellulose aerogel was evaluated using the Ekenstam equation (Equation (3)) [48].

(3) $$k_{t}\;=\;\frac{1}{DP_{t}}-\frac{1}{DP_{0}},$$

where *k_t_* is the rate of cellulose chain scission at time *t, DP_t_* is DP at time *t*, and *DP*_0_ is DP at the initial time *t* = 0. The time (*t_H_*) to reach a 50% loss of DP (*DP_H_*, half-life DP) was evaluated using the rate of cellulose chain scission *k* value derived from Equation (3) as follows:

(4) $$t_{H}=\frac{\frac{1}{\left(DP_{H}\right)}-\frac{1}{DP_{0}}}{k}=\frac{\frac{1}{\left(DP_{0}/2\right)}-\frac{1}{DP_{0}}}{k}=\frac{1}{DP_{0}k},$$

where *t_H_* is the time to reach a 50% loss of DP, and *DP_H_* is half the initial DP (*DP_H_* = *DP*_0_/2).

### 2.5. Brunauer–Emmett–Teller (BET) Surface Area Analysis

The pore characterization of cellulose aerogels was performed by measuring the BET surface area, pore size, and isothermal adsorption curve using a BET Surface Area and Pore Size Analyzer (BELSORP–mini II, MicrotracBEL, Osaka, Japan). BET analysis of cellulose aerogels was performed after absorbing N_2_ gas at 77 K, and the pressure inside the sample cell was varied from 0 to 1 bar. The N_2_ gas adsorption amount, pore volume, and size were measured according to relative pressure.

## 3. Results and Discussion

### 3.1. Solubility Characteristics of Cellulose

**Figure 1:**
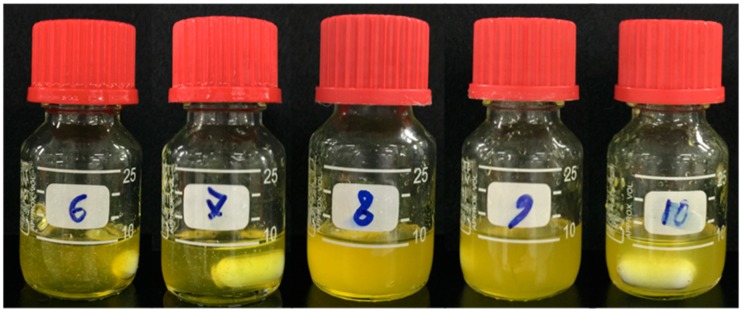
Cellulose solution dissolved in calcium thiocyanate as a function of contained water molecules (From left, in order: 6, 7, 8, 9, and 10 hydrates; Whatman No. 1 paper, 30 min at 120 °C).

In this study, the dissolution of cellulose fiber, cotton linter (Whatman No. 1), with a DP of approximately 2500 using calcium thiocyanate resulted in a difference in the number of water molecules in calcium thiocyanate (Figure 1). In the case of calcium thiocyanate solutions containing six and seven hydrates, the cotton linter fibers were completely dissolved until the liquid was transparent, whereas it can be observed that the solutions containing between eight and 10 hydrates were in a state of suspension in which incompletely dissolved fibers were dispersed. Although the calcium thiocyanate solutions containing over eight hydrates also improved in transparency with reaction time, the suspension could not reach complete dissolution. Hoepfener [18] reported that dissolution using calcium thiocyanate is excellent for porosity material production; in particular, calcium thiocyanate of six to 10 hydrates was reported to be suitable for dissolution. Hattori also reported that calcium thiocyanate containing fewer than 10 hydrates can dissolve cellulose under high temperatures [49]. However, in this study, only calcium thiocyanate containing six and seven hydrates dissolved cellulose effectively. Calcium thiocyanate is generally known to solubilize in cotton fibers, bacterial cellulose, and regenerated fibers, regardless of their crystallinity. Therefore, the difference in solubility is likely more affected by the DP than the degree of crystallization upon the dissolution of cellulose. It is necessary to consider that the dissolution characteristics of calcium thiocyanate may also vary depending on the DP of cellulosic raw materials.

**Figure 2:**
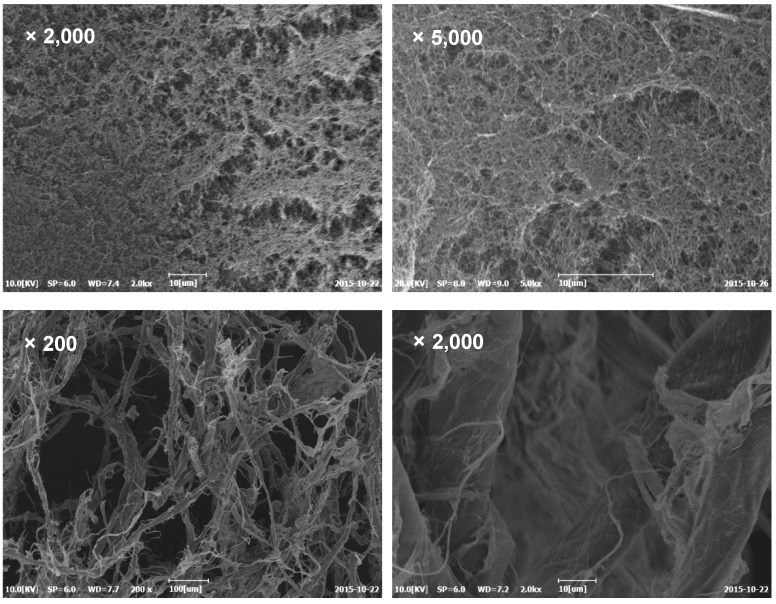
Scanning electron microscope images of cellulose aerogels (**upper**: dissolved in cellulose dissolution of calcium thiocyanate with six (**left**) and seven (**right**) hydrates, **lower**: undissolved in cellulose dissolution of calcium thiocyanate with nine (**left**) and 10 (**right**) hydrates).

The microstructures of the final cellulosic aerogels according to the calcium thiocyanate solution of the different hydrates were compared (Figure 2). In the case of cellulose aerogels dissolved with calcium thiocyanate of six and seven hydrates, relatively uniform microporous structures were confirmed. In contrast, as the number of hydrates was increased, such as with calcium thiocyanate of nine and 10 hydrates, irregular structures and incompletely dissolved fibers were confirmed. Ca^2+^ in calcium thiocyanate has four sites that can coordinate with hydroxyl groups, except for the sites of the two SCN^−^ ions. Therefore, in the case of calcium thiocyanate with over nine hydrates, the complete dissolution of cellulose is difficult because the water molecules interfere with the coordination bond between Ca^2+^ and the hydroxyl group in the cellulose molecules [49]. Therefore, the calcium thiocyanate solution containing a high number of hydrates is considered relatively disadvantageous in completely dissolving the cellulose raw materials, such as those with DPs above 2500. However, if the number of hydrates of the inorganic salt is excessively low, it could be advantageous for cellulose dissolution, but this can excessively increase the viscosity of the cellulose solution, which may make it difficult to apply to the cellulose aerogel.

### 3.2. Evaluation of Cellulose Hydrolysis during Dissolving Process

**Figure 3:**
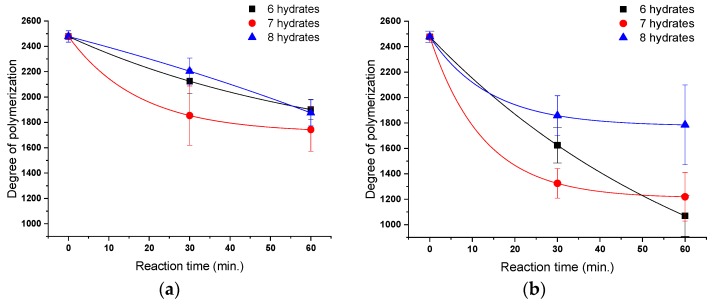
Changes in degree of polymerization (DP) of cellulose (Whatman No. 1 paper) as function of the number of hydrates in calcium thiocyanate (reaction temperature: (**a**) 100 °C, (**b**) 120 °C).

To evaluate the cellulose degradation according to the dissolving conditions in calcium thiocyanate, such as reaction temperature, reaction time, and number of hydrates, absolute molecular weights were measured to compare the DPs. Figure 3 shows the changes in cellulose DP according to the reaction time and temperature with calcium thiocyanate. Although the DP continuously decreased with increasing reaction time, most of the decrease in DP occurred within 30 min of the reaction, and, when the DP reached about 2000, there was no further significant decrease.

In the case of the effect according to the reaction temperature, the DP of cellulose dissolved at 100 °C for 1 h was reduced by about 25%, but that of cellulose dissolved at 120 °C decreased by about 45%, which is about twice as fast as that in the case at 100 °C. In the case of calcium thiocyanate with six and seven hydrates in which cellulose was well dissolved, there was a significant decrease in DP, whereas, in the case of calcium thiocyanate with eight hydrates, which did not completely dissolve cellulose, the reduction in DP was smaller than in that with six and seven hydrates. The decrease in cellulose DP during the dissolving process is considered the result of the reaction of calcium thiocyanate with cellulose in the dissolution process rather than the cellulose autohydrolysis at high reaction temperature. The result of a faster decrease in DP as the hydrate content in calcium thiocyanate decreased is supported by this hypothesis. Cellulose dissolution using inorganic salts proceeds in the following order: (1) dissolution of inorganic salts, (2) swelling of cellulose, and (3) dissociation of cellulose bundles [49]. As the concentration of the inorganic salt increases, the number of inorganic cations bound to the hydroxyl group of cellulose increases, thereby increasing the possibility of eliminating the hydrogen bonding between the cellulose molecules. As a result, it was considered that, as the concentration of calcium thiocyanate increased, the solubility of cellulose increased, whereas the DP decreased with the increasing degree of side reactions generated during the reaction between cellulose and the Ca^2+^ ion.

**Figure 4:**
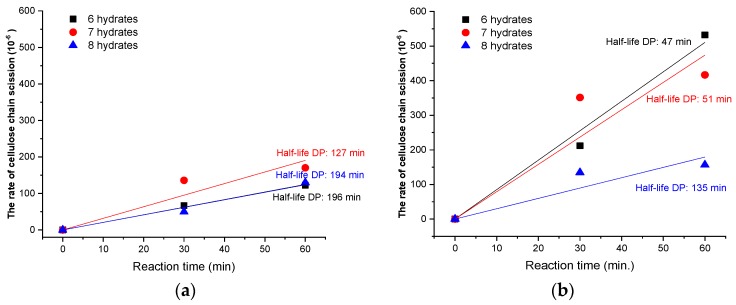
Rate of cellulose chain scission as a function of the number of hydrates in calcium thiocyanate at 100 °C (**a**) and 120 °C (**b**).

To quantify these effects, the kinetics of the cellulose hydrolysis rate according to each reaction condition was evaluated (Figure 4). As a result, it was confirmed that hydrolysis proceeded more rapidly as the number of hydrates decreased. The rates of cellulose chain scission in calcium thiocyanate with six and seven hydrates were 2.08 × 10^−6^ and 3.18 × 10^−6^ min^−1^ at 100 °C, respectively. As the temperature increased to 120 °C, the rates increased to 8.51 × 10^−6^ and 7.90 × 10^−6^ min^−1^, respectively. The rates of cellulose chain scission increased by about 310% and 150%, respectively, as the reaction temperature of calcium thiocyanate with six and seven hydrates increased from 100 to 120 °C. As a result, the half-life DPs were significantly reduced from 196 and 127 min to 47 min and 51 min, indicating that more rapid hydrolysis of cellulose occurred with increasing temperature. However, in the case of the reaction with calcium thiocyanate with eight hydrates, the rates of cellulose chain scission were 2.06 × 10^−6^ and 2.99 × 10^−6^ min^−1^ at 100 and 120 °C, respectively; the reaction rate only increased by 50% and the half-life DP was only reduced from 194 to 135 min by increasing the temperature. This may be the result of the incomplete dissolution of cellulose in the reaction with calcium thiocyanate with eight hydrates. As the temperature was increased by 10 °C, the rate of cellulose chain scission increased by about 2.0, 1.6, and 1.2 times for the calcium thiocyanate with six, seven, and eight hydrates, respectively. From the kinetics of cellulose hydrolysis, the rate of hydrolysis increased as the number of hydrates in calcium thiocyanate decreased and as the reaction temperature increased. Both the number of hydrates in calcium thiocyanate and the reaction temperature are factors that improve the solubility of cellulose. However, to dissolve cellulose with less of a reduction in DP, it is necessary to consider a higher hydrolysis rate increase, owing to the increase in the hydrolysis rate with the decrease in the number of hydrates in calcium thiocyanate rather than a reaction temperature increase.

### 3.3. Evaluation of Cellulose Oxidation during Dissolving Process

**Figure 5:**
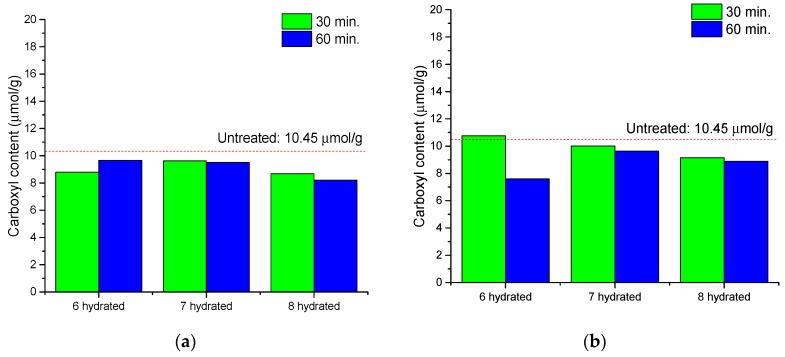
Carboxyl content of cellulose after dissolving as a function of the number of hydrates in calcium thiocyanate at 100 °C (**a**) and 120 °C (**b**).

**Figure 6:**
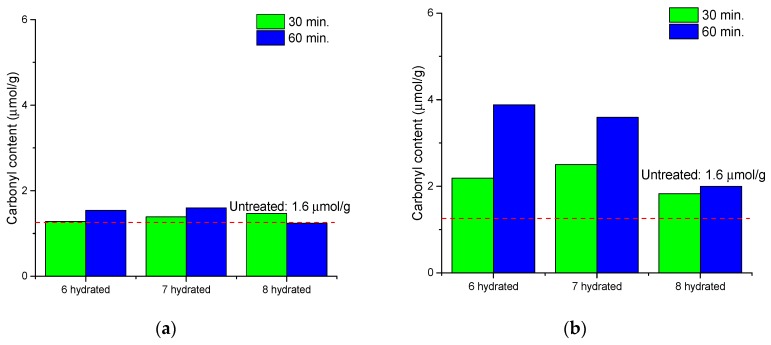
Carbonyl content of cellulose after dissolving as a function of the number of hydrates in calcium thiocyanate at 100 °C (**a**) and 120 °C (**b**).

To evaluate the oxidation of cellulose by calcium thiocyanate, the content of carboxyl and carbonyl groups in cellulose molecules was measured. As shown in Figure 5 and Figure 6, the content of carbonyl and carboxyl groups after the reaction was not significantly different from that before the reaction; only a micromolar increase in the carbonyl group was observed in the cellulose reacted at 120 °C. There was no significant increase in the oxidation of cellulose during the dissolving process. As a result, it is considered that the oxidation reaction is not the main factor affecting the cellulose degradation during the dissolution process using calcium thiocyanate, whereas hydrolysis is considered a major factor in the degradation of cellulose during the dissolving process.

### 3.4. BET Surface Area Analysis

**Figure 7:**
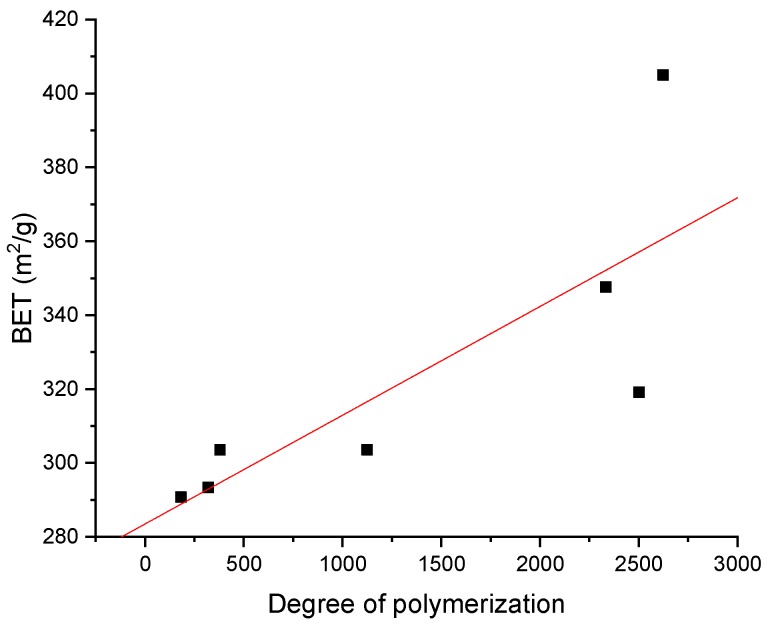
Brunauer–Emmett–Teller (BET) specific surface area of cellulose aerogels as a function of the DP.

**Figure 8:**
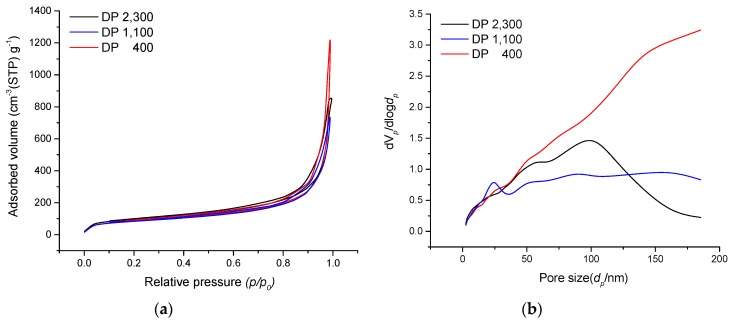
Nitrogen adsorption–desorption isotherms (**a**) and pore size distribution (**b**) of cellulose aerogels with different DPs.

Our previous studies reported that the density, microstructure, and mechanical strength properties of cellulose aerogels were improved by increasing the DP of cellulose molecules [42,43,50]. The BET specific surface areas of cellulose aerogels were measured with nitrogen gas adsorption analysis, as shown in Figure 7 and Figure 8, and it was confirmed that the BET specific surface area was influenced by the DP of cellulose. The observed BET specific surface areas were between 290 and 410 m^2^/g, which is in the typical range of cellulose aerogels [21,51]. The isotherms were classified as IUPAC (International Union of Pure and Applied Chemistry) type IV with a hysteresis loop in the range of 0.1–1.0 P/P_0_, which indicated the presence of meso- and macroporous structures [52,53]. The pore size distribution was found to increase the distribution of macropores as the DP of cellulose decreased. These results explain why the BET specific surface area values decreased with decreasing DP of cellulose. Therefore, it is important to minimize the reduction in the DP of cellulose during the dissolution process of cellulose.

## 4. Conclusions

In this study, the cellulose degradation during the dissolution process using calcium thiocyanate was evaluated. As the number of hydrates decreased, the solubility of cellulose was improved, whereas hydrolysis also increased. The increase in the reaction temperature with calcium thiocyanate improved the solubility of cellulose but also increased cellulose hydrolysis. Therefore, to improve the solubility of cellulose using calcium thiocyanate hydrate, it is necessary to decrease the number of hydrates or to increase the reaction temperature. The problem, however, is that both cases increase the hydrolysis of the cellulose. To minimize the loss of cellulose DP during the dissolution process, it was considered more advantageous to decrease the number of hydrates in calcium thiocyanate than to increase the reaction temperature of the dissolution process. From the measurement of the carboxyl and carbonyl groups to evaluate the oxidation effects during the dissolution process, the influence of cellulose oxidation by calcium thiocyanate was relatively small compared to that of hydrolysis. Therefore, the calcium thiocyanate-induced hydrolysis of cellulose, which can reduce the mechanical properties of cellulose aerogels owing to a decrease in cellulose DP during the dissolution process, was considered a side effect.

To improve the properties of cellulosic aerogels, various studies were conducted on cellulose solvents, solvents for minimizing shrinkage during the wet gel formation process, and the drying method for minimizing the shrinkage of cellulose aerogels during the final drying process. However, in addition to these factors, it should be noted that the cellulose degradation occurring during the cellulosic dissolution process can affect the quality of the final cellulose aerogels.

This study only noted that the BET value of cellulose aerogels could decrease during the dissolution process due to hydrolysis. We expect that the mechanical and dimensional stability of cellulose aerogels can be improved if the DP loss is minimized. This study provides insight for the further development of cellulose aerogels for high-performance applications.
